# Liquid Biopsy Preservation Solutions for Standardized Pre-Analytical Workflows—Venous Whole Blood and Plasma

**DOI:** 10.1007/s40139-018-0180-z

**Published:** 2018-10-18

**Authors:** Daniel Grölz, Siegfried Hauch, Martin Schlumpberger, Kalle Guenther, Thorsten Voss, Markus Sprenger-Haussels, Uwe Oelmüller

**Affiliations:** QIAGEN GmbH, Research & Development, QIAGEN Strasse 1, 40724 Hilden, Germany

**Keywords:** Liquid biopsy, Circulating cell-free DNA (ccfDNA), Circulating tumor cells (CTC), Extracellular vesicles, Exosomes, pre-analytics, Pre-analytical workflows, standardization

## Abstract

**Purpose of Review:**

Liquid biopsy analyses based on circulating cell-free nucleic acids, circulating tumor cells or other diseased cells from organs, and exosomes or other microvesicles in blood offer new means for non-invasive diagnostic applications. The main goal of this review is to explain the importance of preserving whole blood specimens after blood draw for use as liquid biopsies, and to summarize preservation solutions that are currently available.

**Recent Findings:**

Despite the great potential of liquid biopsies for diagnostics and disease management, besides non-invasive prenatal testing (NIPT), only a few liquid biopsy applications are fully implemented for routine in vitro diagnostic testing. One major barrier is the lack of standardized pre-analytical workflows, including the collection of consistent quality blood specimens and the generation of good-quality plasma samples therefrom. Broader use of liquid biopsies in clinical routine applications therefore requires improved pre-analytical procedures to enable high-quality specimens to obtain unbiased analyte profiles (DNA, RNA, proteins, etc.) as they are in the patient’s body.

**Summary:**

A growing number of stabilizing reagents and dedicated blood collection tubes are available for the post-collection preservation of circulating cell-free DNA (ccfDNA) profiles in whole blood. In contrast, solutions for the preservation of circulating tumor cells (CTC) that enable both, enumeration and molecular analyses are rare. Solutions for extracellular vesicle (EV) populations, including exosomes, do not yet exist.

## Introduction

Circulating cell-free DNA (ccfDNA) was first described by Mandel and Metais in 1948 [1]. Later, elevated levels of ccfDNA were found to be associated with pathophysiological conditions and diseases such as systemic lupus erythematosus, rheumatoid arthritis, and certain cancer types (reviewed in [2]). Stroun and coworkers [3] were the first to identify ccfDNA originated from tumors, which eventually led to the term “liquid biopsy” for the use of blood plasma for purification and analysis of tumor-derived DNA. Since then, it has been demonstrated that circulating tumor DNA (ctDNA) can be analyzed to investigate or monitor pathological changes in the tumor genome—from chromosomal aberrations such as microsatellite alterations, rearrangements, amplifications, and copy number variations—down to single nucleotide exchanges and epigenetic changes (reviewed in [4•]). ctDNA in whole blood reflects the tumor genome heterogeneity, including primary tumor and distant metastasis. Analyzing ctDNA for diagnosis and therapy monitoring could be a way for non-invasive early detection of new upcoming or resistance-mediating driver mutations [4•].

Existence of cell-free fetal DNA (cffDNA) circulating in the maternal bloodstream was initially published by Lo and colleagues [5]. They were also among the first to recognize the potential of using ccfDNA for noninvasive prenatal testing (NIPT), such as determination of rhesus factor [6], and detection of aneuploidy [7] and single gene disorders [8]. For NIPT, cffDNA analyses have meanwhile become routine clinical practice. For example, Denmark and The Netherlands have implemented nationwide prenatal screening for fetal RhD-positive maternal plasma based on quantitative PCR (qPCR) [9]. Analysis of fetal chromosomal aberrations is offered by numerous companies and is already implemented or will be implemented in the healthcare systems of many countries. In addition, ccfDNA analysis has been described as a potential complement to existing diagnostic approaches for numerous other clinical areas, like myocardial infarction, sepsis, trauma, diabetes, and transplantation medicine [2, 10].

The major source for ccfDNA are mono-nucleosomal DNA fragments originating from apoptotic and necrotic cells [11]. Furthermore, extracellular DNA is also present as vesicle-bound apoptotic bodies, microparticles, microvesicles, exosomes or histone/DNA complexes, nucleosomes, and virtosomes [12, 13]. In addition, cell-free RNA is present inside exosomes and other extracellular vesicles (EVs). EVs have been shown to contain various small RNA species, including miRNA, piRNA, tRNA (and fragments thereof), vault RNA, Y RNA, fragments of rRNA, as well as long non-coding RNA, and apparently fully intact mRNA [14, 15]. From a clinical perspective, ccfRNA analysis appears promising for disease detection and patient stratification and monitoring, particularly with respect to detection of fusion transcripts and splice variants.

In contrast to tumor-derived circulating nucleic acids and exosomes, circulating tumor cells (CTCs) are considered “living” liquid biopsy species of cancer that most likely represent the seeds of metastasis [16]. CTCs are tumor-derived cells that circulate in cancer patients’ blood, in contrast to disseminated tumor cells (DTCs), which are found in bone marrow. Morphologically, CTCs are often described as epithelial-like cells expressing cytokeratins and epithelial markers like EpCAM [17]. These cells can be differentiated from PBMCs because they are usually larger than leukocytes and negative for CD45 [18]. More recently, other subtypes of CTCs have been described which lose their epithelial origin and undergo epithelial to mesenchymal transition (EMT) and tumor stem-like phenotype changes. Such cells are potentially negative for cytokeratins but express EMT markers [19, 20]. These EMT or stem cell-like CTCs are discussed to represent a therapy-resistant subtype that correlates to metastasis formation [21].

Since Cristofanilli et al. [22] demonstrated the prognostic meaning of CTCs in metastatic breast cancer using the CellSearch device (Menarini Silicon Biosystems, Castel Maggiore, Italy) for CTC enumeration, such prognostic value has been demonstrated for several solid cancer entities like prostate, ovarian, and colorectal cancer [23]. In addition to enumeration, it is meanwhile well-known that further molecular characterization of CTCs provides valuable information about the CTC metabolism in the context of therapy resistance mechanisms [24••, 25•] and phenotype changes like EMT and tumor stem cell formation [26]. CTCs reflect the tumor complexity that emerges during tumor progression. In addition to the prognostic value of CTC identification and counting in cancer progression, molecular characterization of CTCs can guide treatment decisions and improve patient outcome.

## Preservation Solutions for Improving and Standardizing Pre-Analytical ccfDNA Workflows for Liquid Biopsy Analyses

### Known and Unknown about ccfDNA

**Fig. 1:**
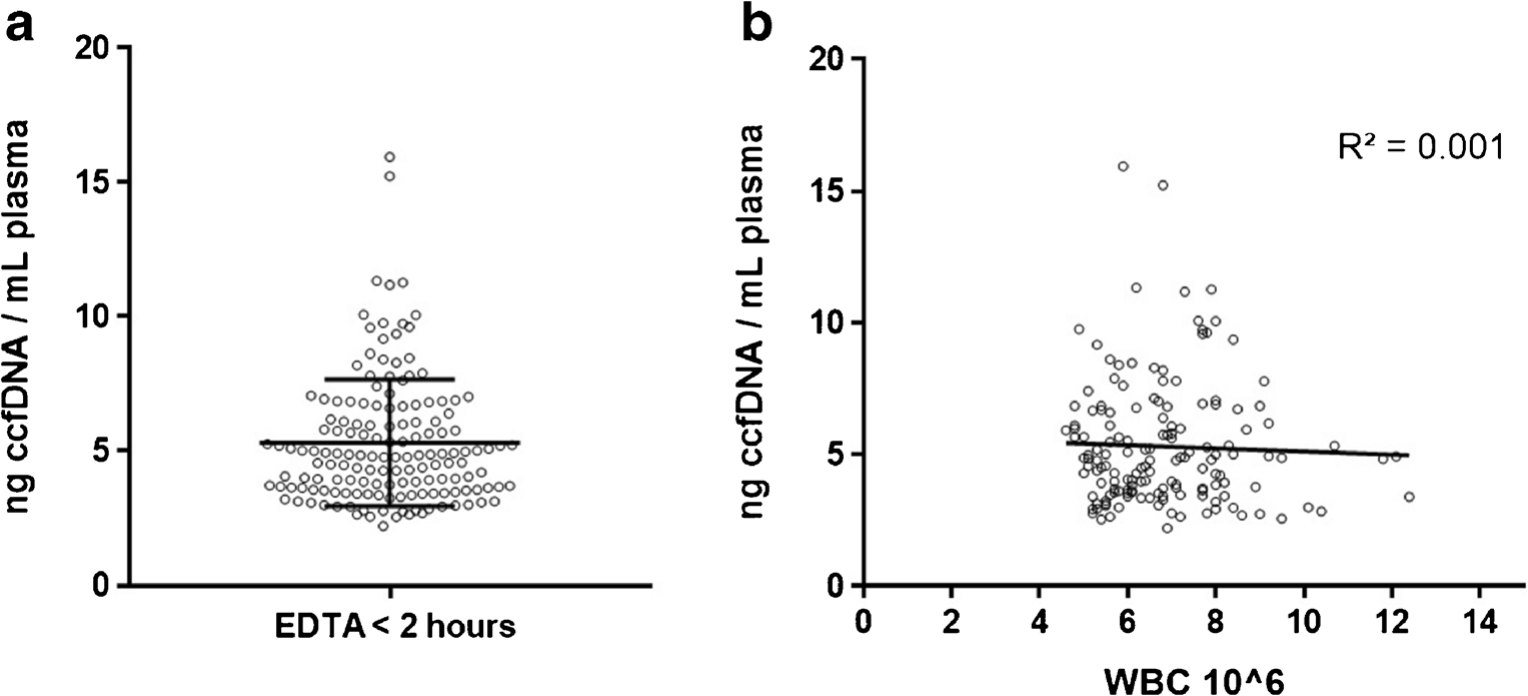
ccfDNA yield in K2-EDTA blood and correlation to white blood cell (WBC) count. **a** Yield of ccfDNA per milliliter plasma from K2-EDTA blood quantified with Qubit 2.0 (Thermo Fisher). Blood samples from 152 healthy donors were processed within 2 h after venipuncture and ccfDNA isolated with the QIAsymphony DSP Circulating DNA Kit (QIAGEN); depicted is the scatter plot with mean and standard deviation. **b** Correlation between ccfDNA yield per milliliter EDTA plasma plotted and WBC count; depicted are trend line and *R*-squared value

Although ccfDNA has been investigated for a long time in many applications, surprisingly, few details are known about its origin and function, both under physiological and pathophysiological conditions. In healthy donors, ccfDNA is usually of low concentration in plasma with a median of about 5 ng/ml, but differs between individuals (Fig. 1a). In general, increased ccfDNA content in blood can be seen as an indicator of unusually high cell death linked to different pathological conditions [10, 27]. In cancer, the concentration of ctDNA can vary drastically, depending on tumor size, stage, location, and other factors, with a proportion of ctDNA between 0.01 and 90% (reviewed in [4•]). High ctDNA load can be associated with poor prognosis (reviewed in [28]). There is some disagreement about the size of ctDNA. In most reports, ctDNA is described as more fragmented compared to ccfDNA from normal tissues [29, 30]. This factor might even be used for enrichment [29]. Others, on the contrary, found that integrity of certain tumor-derived fractions increased in some cancer types [31, 32]. The predominate size of about 166 bp [29, 33–35] corresponding to the size of mono-nucleosomal DNA fragments supports the assumption that apoptosis is the major source for ccfDNA [11]. Longer circulating DNA might also occur especially in the case of cancer patients, originating from necrosis of tissue surrounding the tumor [35].

In pregnant women, cffDNA is supposedly derived from trophoblasts in the placenta [36], but according to Lui et al. [37] and others, the main sources for ccfDNA in healthy subjects are hematopoietic cells with the DNA deriving from apoptotic but not necrotic cells [38]. However, if blood cells were the main source for ccfDNA in healthy subjects, there should be a correlation between white blood cell (WBC) count and ccfDNA yield, which we were unable to find, analyzing blood samples from more than 150 healthy subjects (Fig. 1b). Others have reported that all living cells actively release DNA into circulation [39, 40]. Extracellular DNA might be part of a physiological pathway for horizontal gene transfer between distant cells [12]. The uptake of ctDNA by normal cells in cancer reportedly can trigger distant metastasis, a process called genometastasis [41, 42].

### Pre-Analytical Workflow of ccfDNA

In contrast to NIPT and in spite of its potential and broad application spectrum, analysis of ctDNA from tumor patients has not yet become standard in healthcare but is mostly restricted to biomedical and clinical research. Until now, only two assays for ctDNA analysis have received FDA approval (Epi proColon from Epigenomics, Berlin, Germany, and cobas EGFR Mutation Test from Roche Molecular Diagnostics, Basel, Switzerland). Concordantly, many authors have pointed out the lack of workflow standardization and protocol harmonization for pre-analytics [4•, 43, 44•, 45, 46•, 47]. This shortcoming along with insufficient knowledge about the origin and function of ctDNA are the main reasons why ctDNA analyses have not found their way into clinical routine [46•, 47].

The pre-analytical workflow for analysis of ccfDNA includes all steps from venipuncture to preparation of the isolated ccfDNA for downstream analysis. These workflow steps include blood collection and preservation, blood storage and transport conditions, time elapsed between specimen collection and processing for plasma generation, plasma storage and/or transport conditions, ccfDNA isolation, and storage (Table 1). Each step of the pre-analytical workflow can affect or even falsify the analytical outcome, leading to contradictory study results and reports (reviewed in [46•]). In particular, dilution of ccfDNA with genomic DNA released from apoptotic and lysed leukocytes after blood draw constitutes one of the most prominent challenges in ccfDNA analysis.

**Table 1:** Pre-analytical factors that can influence outcomes of ccfDNA analysis

Pre-analytical step affecting ccfDNA	Challenge	Recommendation	Quote
Blood collection tube	Ex vivo release of genomic DNA from leukocytes; PCR inhibition	Use of a dedicated ccfDNA stabilization tube	[48••]
		Use of EDTA tube, in case no dedicated ccfDNA stabilization tube is available	[49]
Time between blood collection and plasma processing	Ex vivo release of genomic DNA from leukocytes	Must be verified and validated in combination with downstream application In case of unstabilized EDTA blood, processing should be performed within 2 to 6 h	[44•, 48••, 50, 51•]
Plasma or serum	Ex vivo release of genomic DNA from leukocytes	Use of plasma	[48••, 52, 53, 54]
Plasma processing protocol	Incomplete separation of cellular fraction; mechanical lysis of blood cells	For EDTA blood, use double centrifugation protocol with low and high speed centrifugation Follow ccfDNA tube manufacturer’s instructions	[44•, 48••, 55, 56]
Plasma storage	Reduction of yield; increased fragmentation	Do not store plasma at 2–8 °C for longer than 24 h Freeze at − 20 °C or − 80 °C Avoid repeated freezing/thawing cycles	[44•, 48••, 51•]
DNA purification method	Suboptimal compatibility with blood collection tube; low yield due to DNA loss; isolated ccfDNA lengths bias	Validation and verification of DNA purification method Use of an integrated system of kit and tube Follow ccfDNA tube manufacturer’s instructions	[43, 48••, 58–60]
DNA quantification	Over quantification due to impurities and detection limit in spectrophotometry	Use of qPC- based methods rather than spectrophotometry	[43, 48••, 59]
DNA storage	Reduction of yield Increased fragmentation	Store ccfDNA at − 20 °C or below Avoid repeated freezing and thawing	[48••, 51•]

Via the European Committee for Standardization (CEN) and its specific Technical Committee 140 for in vitro diagnostic medical devices (CEN/TC 140), the European Union’s FP7 consortium project SPIDIA (www.spidia.eu) initiated for the first time pan-European evidence-based standard documents for covering all pre-analytical workflow steps for dedicated molecular in vitro diagnostics. Nine such standard documents were released as CEN Technical Specifications (CEN/TS) in European countries by their National Standard Bodies in 2015. Eight of these are expected to become ISO International Standards in 2018 or 2019, including that for ccfDNA pre-analytical workflows. In the specific CEN/TS for ccfDNA pre-analytical workflows, usage of dedicated blood collection tubes with a ccfDNA stabilization reagent is recommended [48••]. In the event that no ccfDNA stabilization collection tube is available, the CEN/TS as well as Lam et al. [49] suggest that EDTA is preferred as anticoagulant over citrate and heparin because of a more moderate release of genomic DNA within the first 24 h after blood collection. Most authors recommend plasma processing from EDTA anticoagulated blood within 2 to 6 h after collection [44•, 49, 50, 51•], and the consensus is that plasma is preferred over serum for purification of ccfDNA because of ex vivo release of DNA during the clotting process [51•, 52–54].

The plasma generation protocol also has an impact on ccfDNA yield [44•, 55, 56]. Two consecutive centrifugation steps to generate plasma are applied in many if not most laboratories, a procedure that dates back to Chiu et al. [55]. Modifications to this protocol may be required if a stabilization reagent such as formaldehyde is added to the blood specimen [57•] or when a dedicated blood collection tube with a stabilization reagent is used. Once plasma is separated, it should be stored frozen at − 20 °C or even at − 80 °C and repeated freeze-thaw cycles should be avoided [44•, 51•].

The method of extraction can have a profound impact on DNA yield, which is crucial for, e.g., the detection of rare somatic mutations or correct quantification [43, 45, 58–60]. In general, verification and validation of the ccfDNA isolation method is a required component of the verification and validation of the whole diagnostic workflow. In case that a dedicated collection and stabilization tube is used, manufacturer’s recommendations should be followed [48••], which can for instance include a prolongation of proteinase K digestion steps if a blood collection tube with a stabilization reagent that leads to cross-linked molecules is used. For quantification, qPCR-based methods are preferred over spectrophotometry because impurities might interfere with spectrophotometrical measurements and their reliability usually declines in the lower DNA concentration range [45]. Finally, isolated ccfDNA should be stored frozen at − 20 °C or below and repeated freeze-thaw cycles should be avoided [51•].

### Stabilization Solutions for ccfDNA

The challenge of rapid ex vivo release of genomic DNA in native blood after collection requires preservation of blood cells. In case that logistics do not allow to tightly control storage durations and conditions between phlebotomy and plasma generation, use of a fixative or a dedicated blood collection tube with a ccfDNA stabilization reagent is recommended [48••]. This is especially the case in clinical trials or in clinical routine whenever blood samples are collected at external sites, away from the molecular laboratory, and blood processing for plasma generation at the point of collection is not feasible.

Dhallan et al. [57•] proposed to preserve maternal blood cells by adding neutral buffered formaldehyde (NBF) to EDTA-anticoagulated maternal blood directly after blood collection in combination with a gentle centrifugation protocol. They suggested that formaldehyde reduces cell lysis through cell membrane stabilization and inhibition of nucleases, and reported a substantial increase of the relative percentage of fetal-derived cffDNA over total ccfDNA. Despite no agreement about this increase (confirmation by Benachi et al. [61], contradiction by Chung et al. [62] and Chinnapapagari et al. [63]), the stabilizing effect of formaldehyde to impede cell lysis is accepted and occasionally employed for analysis of plasma DNA [64]. But the need to open each blood collection tube directly after phlebotomy to add formaldehyde is an additional handling step that is difficult to standardize and implement at specimen collection sites. Furthermore, formaldehyde is toxic [65], and because of its potential carcinogenicity, formalin was recently reclassified in the EU (category 2/3 to category 1B/2) and must now be labeled as carcinogenic and mutagenic [66]. Formaldehyde’s mode of action is crosslinking and chemical modification of biomolecules, which leads to non-reproducible DNA sequence alterations or loss of PCR products [67].

Streck Inc. (Omaha, USA) was the first to include a cell-stabilizing reagent into a blood collection tube (BCT), the Cell-Free DNA BCT (Streck Tube), intended to stabilize ccfDNA levels by preventing gDNA release from blood cells [68••]. Following Streck’s published intellectual property, the active component in the BCT reagent is most likely a formaldehyde-releasing substance like diazolidinyl urea (US 2010/0209930 A1, US 2010/0184069 A1) and K3-EDTA as anticoagulant. Streck reported that free formaldehyde cannot be detected in the BCT reagent by carbon-13 nuclear magnetic resonance analysis [69] and that the reagent neither causes damage to DNA nor has any negative effect on DNA amplification [70]. Nevertheless, components within the BCT reagent react with semi-quantitative MQuant Formaldehyde test strips (EMD Millipore Corporation, MA, USA) and the size profile of ccfDNA shows a shift toward higher molecular weight compared to unmodified ccfDNA from EDTA blood when analyzed on the Agilent Bioanalyzer (unpublished results). This shift could indicate chemical DNA modification by a crosslinking substance which was not reverted during DNA isolation.

The Streck Tube was evaluated for NIPT applications by different groups. Hidestrand et al. [71] found no significant difference in total maternal ccfDNA from Streck Tubes shipped for 72 h at room temperature versus matched samples from EDTA blood processed immediately. They noted, however, an increase in total ccfDNA and consequently a significant decrease in the cffDNA fraction in samples shipped at lower temperatures with cool packs. Wong et al. [72] confirmed that Streck Tubes stabilize cell integrity and the cffDNA fraction in maternal blood, but they also reported a post-collection increase in total ccfDNA yield after 14 days storage at room temperature or 1 day at elevated temperatures of 37 °C to 40 °C. Usability of Streck Tubes for mutation detection by qPCR in cancer-related applications was shown by Denis et al. [73] for melanoma and by Sherwood et al. [74] for NSCLC patients using the *therascreen* BRAF and KRAS RGQ PCR Kits (QIAGEN, Hilden, Germany), respectively. More recently, mutation detection with ctDNA from Streck Tubes was demonstrated with droplet digital PCR (ddPCR) by Sacher et al. [75] and with BEAMing and Safe-Sequencing by Diaz et al. [76]. Some unclarity exists about the compatibility of the stabilization technology in Streck Tubes with epigenetic tests requiring bisulfite treatment prior to qPCR. Schmidt et al. [77••] mentioned that the Streck Tube does not seem to work for this application. According to Distler et al. [78], methylated SEPT9 colorectal cancer screening marker can be detected with almost 100% sensitivity in blood from EDTA tubes stored at 2–8 °C for a maximum of 24 h, as well as in blood from CPDA tubes stored at 18–25 °C for up to 48 h using the Epi proColon (Epigenomics, Berlin, Germany) colorectal cancer screening test. Sensitivity dropped to 7% when using blood from Streck Tubes stored for 7 days at 25–30 °C.

In 2016, PreAnalytiX (Hombrechtikon, Switzerland) launched the integrated PAXgene Blood ccfDNA System. This system consists of the PAXgene Blood ccfDNA Tube (PAXgene Tube) and a ccfDNA purification kit for automated extraction of ccfDNA on the QIAsymphony SP instrument (QIAGEN) or manual extraction using the QIAamp Circulating Nucleic Acid Kit (QIAGEN). According to the manufacturer, the stabilization reagent in the PAXgene Blood ccfDNA Tube prevents blood coagulation, lysis of red blood cells, and apoptosis of white blood cells. The stabilization reagent in the tube is free of cross-linking or cross-linker-releasing substances (www.preanalytix.com) and hence does not chemically modify ccfDNA. Schmidt et al. [77••] evaluated the PAXgene Blood ccfDNA System, including manual and automated extraction, for the quantification of methylated mSHOX2 plasma DNA in lung cancer patients. They found that total yield of ccfDNA was stabilized in PAXgene tubes when samples were stored for 7 days at room temperature and quantification of methylated mSHOX2 sequence by qPCR following bisulfite treatment was possible. Warton and colleagues [79•] compared K2-EDTA, Streck, and PAXgene tubes for ccfDNA stabilization and fragment size. They found that in contrast to EDTA, both Streck and PAXgene tubes stabilized ccfDNA level when stored for 4 days at room temperature. They noticed contamination with DNA of high molecular weight in Streck Tubes after 4 days of storage, which did not change the ratio between a long and short amplicon of Alu sequences determined by qPCR. They speculated that this high molecular weight DNA in Streck Tubes contains chemical modifications such as cross-linked proteins and is therefore less amenable to PCR.

Concurrently, with PreAnalytiX, Ariosa Diagnostics, Inc. (San Jose, USA, acquired by Roche Diagnostics, Basel, Switzerland) launched the Cell-Free DNA Collection Tube (Roche Tube). Information about the composition of the proprietary reagent in this tube is not available. Recently, two groups independently published comparison tests using Streck, PAXgene, and Roche tubes. Alidousty et al. [80•] spiked blood directly after venipuncture with DNA of the T790M mutated EGFR gene fragmented by sonication. After 7 days storage at room temperature, they were able to detect mutated DNA equivalent to 40 gene copies per milliliter plasma from all tubes by qPCR. When the spike-in was reduced to approximately 20 gene copies per milliliter plasma, only PAXgene and Roche tubes allowed reliable detection of the mutated DNA after 1 day of storage. In contrast, Nikolaev et al. [81•] found the performance of PAXgene and Streck tubes to be superior to that of Roche tubes when compared in a time course validation study. Whereas PAXgene and Streck tubes efficiently prevented plasma contamination with genomic DNA when blood was stored for 7 days with temperature cycles between 22 °C and 30 °C, high molecular weight DNA appeared in Roche tubes after 5 days, as determined by qPCR.

In 2017, additional dedicated blood collection tubes with proprietary ccfDNA stabilizing reagents were launched, but these have not gained attention in peer-reviewed journals so far (Table 2).

**Table 2:** Blood collection tubes with dedicated ccfDNA stabilization reagent

Blood collection tube	Manufacturer	Draw volume	References
Cell-Free DNA BCT®	Streck, Omaha, USA	10.0 ml	[54, 68••, 70–76, 78, 80, 81•]
PAXgene® Blood ccfDNA Tube	PreAnalytiX GmbH, Hombrechtikon, Switzerland	10.0 ml	[77••, 80•, 81•]
cfD Tube	Roche Diagnostics (Schweiz) AG, Basel, Switzerland	8.5 ml	[80•, 81•]
LBgard™ Blood Tube	Biomatrica, Inc., San Diego, USA	8.5 ml	–
Blood Stasis™ 21-ccfDNA Tube	Mabio Genomics, Inc., Gaithersburg, USA	9.0, 6.0, 3.0 ml	–
cf-DNA Preservative Tube	Norgen Biotek, Corp., Thorold, Canada	8.3 ml	–
Nice® Check cfDNA Tube	EONE-DIAGNOMICS Genome Center, Incheon, Korea	8.0 ml	–
Blood Exo DNA ProTeck® Tube	CFGenome LLC, Denver, USA	nm	–
ImproGene Cell Free DNA Tube	Improve Medical Instruments Co., Ltd., Guangzhou, China	10.0 ml	–

## Preservation of Circulating Tumor Cells

### CTC Isolation for Enumeration and Staining

CTCs are a very rare cell population with 0–500 cells per 10 ml blood. Especially in early disease, they can, e.g., amount to less than 10 cells per 10 ml blood, representing a ratio of approx. 1:10^7^ CTCs to WBCs. Therefore, pre-enrichment seems to be required for most analytical downstream assays. Current technologies for enrichment of CTCs include antibody-based enrichment methods like AdnaTest (QIAGEN, Hilden, Germany), CellSearch System (Menarini Silicon Biosystems, Castel Maggiore, Italy), and Gilupi CellCollector (Gilupi, Potsdam, Germany), as well as label-free enrichment methods based on physical properties of CTCs like size, deformability, and adhesion (ClearCell Fx from Clearbridge BioMedics, Singapore; Parsortix from ANGLE plc, Guildford, UK; and VTX-1 from Vortex BioSciences, Menlo Park, USA). Taking into account low CTC numbers, the risk of post-collection changes in CTC analyte profiles during all workflow steps including enrichment, and the tendency of CTCs to degrade, the importance of proper pre-analytical steps becomes evident. Such steps include sample collection, preservation, transport, and storage, as well as sample preparation including enrichment for the analytical assay. There are differences in pre-analytical preservation requirements of the various downstream analyses performed, such as CTC counting, or CTC transcriptome, genomic, and proteomic analyses by advanced staining.

The only CTC enumeration system that has obtained FDA clearance so far is the CellSearch System consisting of the CellSave preservative blood collection tube (CellSave BCT), the CTC kit, and the autoprep and analyzer instruments. The system is intended to provide independent prognostic information in several cancer entities. However, for prognostic information on disease-free survival and overall survival, CTC counting alone is meanwhile regarded as insufficient for medical decisions and therapy optimization [82]. Instead, a comprehensive molecular characterization of CTCs is increasingly requested, especially with a focus on predictive information that may allow better therapy planning or the identification of actionable molecular candidates for targeted therapy strategies [83].

In CTC counting and staining procedures, cell preservation must ensure cell integrity during blood specimen transport, storage, and sample batching to generate reproducible results [84–86]. Furthermore, intracellular proteins as well as cell surface antigens must be preserved to enable efficient antibody-mediated enrichment and detection by immunofluorescence. Staining procedures for cell counting do not require live cells but can start from non-viable cells only if the CTC morphology is sufficiently preserved and CTC damage and degradation are avoided. The CellSearch system is the most commonly used platform for these applications. The CellSave BCT was developed for the indicated purpose to allow a delay of up to 72 h at room temperature between blood collection and sample processing [87]. Another CTC stabilizer for enumeration is contained in TransFix tubes [88] (Caltag Medsystems, Ltd., Buckingham, UK), claiming to stabilize CTCs for up to 72 h at room temperature when subsequently enriched using the ScreenCell Cyto device (ScreenCell, Westford, USA) followed by whole genome amplification (WGA) and sequencing.

### Molecular Analyses of CTCs

Recent publications have shown that mutational analysis is possible using single isolated CTCs from blood samples stabilized for counting [89•, 90•], as well as using viable CTCs isolated from EDTA blood [89•, 91]. Shaw et al. were able to identify Pi3K, ESR1, P53, and KRAS mutations in breast cancer patients on a single-cell level using a combination of the CellSearch platform, including CellSave BCT samples, and the DEParray instrument [90]. Kidness-Sigal et al. reported mutational analysis with viable CTCs isolated from EDTA blood [91].

For valid, reliable, and reproducible CTC transcriptome analyses, minimizing post-collection ex vivo gene expression changes is essential, as RNA profiles can change significantly by, e.g., gene induction, gene downregulation, and RNA degradation. Using fixatives developed for cell staining and counting (i.e., the CellSave BCT) is inappropriate in this context because they seem to completely inhibit or at least deteriorate access to intact mRNA [92•, 93]. For specific analytical tests using the AdnaTest System (QIAGEN), a commercially available platform for CTC transcriptional characterization of selected tumor-associated genes rather than cell enumeration, ACDA (acid citrate dextrose formula A) is recommended as an anticoagulant with an acceptable stabilizing effect on the specific RNA molecules analyzed by the so far developed assays for at least 30 h transport under cold conditions (4–8 °C; internal data, not shown). Using the AdnaTest in combination with ACDA-anticoagulated blood, Aaltonen et al. demonstrated molecular changes in CTC metabolism under systemic therapy pressure [94]. Luk et al. compared ACD and K2-EDTA tubes, Cell-Free DNA and Cell-Free RNA BCTs (Streck), and Cyto-Chex BCT (Streck) in a prostate CTC setting, analyzing androgen receptor variant 7 (ARv7) with a ddPCR assay after CTC enrichment on the AutoMaCS Pro Separator (Miltenyi, Bergisch Gladbach, Germany) [92•]. They found that the CTC AR v7 transcript remains stable in EDTA or ACD-anticoagulated blood for 48 h at room temperature, whereas mRNA detection in all the three Streck BCT tubes dropped directly after blood collection and was impossible to measure after any transport time. Wong et al. were able to analyze a panel of 26 genes related to the androgen receptor metabolism as well as EMT and proliferation markers from CTCs using EDTA blood stored for 72 h at 4–8 °C. They used a hematologic cell depletion, microfluidic technology called CTC-iChip [95]. The possibility of achieving sufficient CTC preservation for up to 96 h in EDTA tubes followed by flow sorting and RNA profiling was studied by Apostolou et al. [96•]. They reported only minimal alteration in the expression of specific genes (16 genes as well as 18srRNA and 28 srRNA) for up to 72 h under refrigerated conditions (4–8 °C) and positive microscopic detection of CTCs following immunomagnetic enrichment up to 96 h after collection.

However, keeping blood samples under controlled, cooled conditions during the entire pre-analytical workflow, including limiting the time needed for sample collection, transport, and storage prior to processing in the receiving laboratory, is a significant logistical challenge and difficult to standardize. Achieving and verifying a closed cooling chain is time and resource consuming. Furthermore, cooling of samples may not prevent transcript changes for all targets, and may limit the selection of appropriate target transcripts for CTC detection already at the beginning of new CTC assay or biomarker development. Novel stabilization technologies for preserving CTCs and their analyte profiles (e.g., DNA, RNA, proteins) in whole blood across all pre-analytical workflow steps from collection and storage to enrichment are still needed.

## Preservation of Cell-Free RNA, Exosomes, and Other Extracellular Vesicles

Due to high endogenous RNase activity in blood plasma and serum, any cell-free RNA that is not already protected in vivo is degraded very rapidly. While the existence of a stable cell-free population of miRNAs associated with Argonaute proteins [97•] and perhaps other protective proteins [98, 99] has been demonstrated, no such protective proteins have been identified for mRNA and other long RNAs. Thus, these species have been found almost exclusively within EVs. EVs are remarkably stable, both in vivo and after isolation, with the same stability also conferred to their RNA content [100]. Consequently, protection from endogenous RNases is not a problem for the preservation of cell-free RNA profiles. In contrast, the continuous production, release, and uptake of existing EVs by various blood cells is an issue that must be considered whenever there is a substantial delay between blood collection and preparation of plasma or serum ([101] and own unpublished observations). Similarly, apoptosis and necrosis of blood cells after sample collection will result in release of cellular RNA which can lead to unwanted background. While mRNA and other long RNA species released in that way are rapidly degraded by endogenous RNases, miRNA associated with Argonaute proteins can persist in plasma for prolonged periods [97•].

Existing preservation solutions for ccfDNA profiles designed to prevent cell lysis and apoptosis also minimize release of non-vesicular miRNA after blood collection. However, they are not effective in shutting down the release of new EVs from blood cells [own unpublished observations]. Currently, there is no preservation technology published or commercially available to keep the concentration and population of EVs in blood constant after venipuncture. According to IP filings, Cell-Free RNA Blood Collection Tubes from Streck seem to contain aurintricarboxylic acid as an RNase inhibitor. As outlined above, inhibition of RNases is not required to protect existing cell-free RNA, but may slow down degradation of RNA released during or after blood collection by blood cell necrosis or apoptosis, thereby potentially even increasing unwanted background RNA. Thus, in the absence of effective solutions to block EV production and uptake, the best advice remains to process blood samples into plasma as quickly as possible, ideally within 30 min after venipuncture [101, 102•]. Plasma is preferable over serum because, similar to ex vivo release of ccfDNA during the clotting process, coagulation has also been shown to be accompanied by the release of EVs, particularly from platelets [102•, 103, 104].

## Stabilization Solutions for Multi-Modality Applications

The volume of blood that can be drawn from cancer patients during therapy is often tightly restricted. Collection of blood into multiple different tubes with dedicated stabilization solutions for different analytes is therefore limited. A number of groups investigated if the CellSave BCT, currently regarded as the gold standard for CTC enumeration, can also be used for ccfDNA extraction. Kang et al. [105] found the CellSave BCT to stabilize ccfDNA equivalently to EDTA and Streck Cell-Free DNA Tubes for up to 6 h after venipuncture. However, after 48 h storage at room temperature, they also noticed a moderate decrease in yield as determined by ddPCR. Van Dessel et al. [106] reported that ccfDNA yield from CellSave BCT processed within 96 h at room temperature is comparable to Streck Tubes, and that mutation detection and allele frequency determination by ddPCR is not affected by the stabilization reagent. A similar finding was published before by Rothwell et al. who found ccfDNA from CellSave BCT plasma was preserved for up to 96 h at room temperature and suitable for NGS applications [89•]. For a combined analysis of CTC and ccfDNA from 1 sample, they proposed splitting blood from 1 tube (i.e., 7.5 ml for CTC isolation with the CellSearch System and the remaining 1 to 2.5 ml for isolation of ccfDNA). The difficulty with this approach is that both analyses require the highest possible sensitivity. For most applications, splitting a 10 ml blood sample is not likely a viable option. Several providers of blood collection tubes with stabilization reagents for liquid biopsy applications have claims for ccfDNA and CTC preservation (e.g., Streck Cell-Free DNA BCT and Biomatrica LBgard Blood Tube), but as long as there is no convincing preservation and enrichment concept available for parallel isolation of all required liquid biopsy components from 1 blood sample, there seems to be no alternative to using different tubes for different purposes.

## Conclusions

The use of liquid biopsies in routine healthcare and biomarker discovery and development holds great promises. Advances in sequencing technologies have made it possible to reconstruct the whole tumor genome from ctDNA, and molecular characterization of the genome, transcriptome, and proteins of CTCs enables elucidation of tumor complexity and heterogeneity on a single-cell level. Analyses of biomarkers from liquid biopsies have the potential to become an indispensable tool for diagnosis, prognosis, early detection of therapy resistance, and overall cancer patient care. Currently seen as a complementary tool, liquid biopsies might even replace some more invasive techniques such as core needle biopsies in specific situations.

Despite this high potential, until now, liquid biopsies have fully fulfilled expectations only in the field of NIPT. Besides a better understanding of origin and function, improvements and standardization of complete diagnostic workflows will be key for broader use of CTCs, as well as other circulating cells and free-circulating or vesicle-bound nucleic acids, in patient management. The whole diagnostic workflow includes all steps performed such as specimen collection, preservation, transport, storage, processing, analyte enrichment and/or isolation and storage, execution of the analytical assay, data management, and interpretation including bioinformatics. Only if all relevant workflow components and individual steps are fully verified and validated in the context of the intended use of the analytical test, results can have the reliability and statistical power to be the basis for a therapeutic decision.

The indispensable first step of workflow development and its standardization is the collection and preservation of the specimen. As shown by many researchers, analysis of specific molecular targets from liquid biopsies is possible using non-stabilized blood samples, i.e., EDTA-anticoagulated blood. However, post-collection ex vivo changes in sample molecular profile make high levels of pre-analytical workflow controls in terms of steps durations and conditions imperative when working with unpreserved blood. Cooling, sample processing needs at the point of collection, and short transport and storage duration are very challenging and may even be impossible to maintain in routine healthcare settings. Therefore, use of dedicated stabilization solutions seems to be highly recommendable.

Analysis of free-circulating RNA or EV-bound nucleic acids is still mainly restricted to biomedical or clinical research, partly because technical solutions for specimen and sample preservation are not sufficiently available yet. They are urgently needed to move the field toward clinical applications.

Use of CTC preservation reagents, such as in the CellSave BCT, has been shown to stabilize CTCs for enumeration and might become a complementary part of conventional cancer treatment decision-making and therapy monitoring. However, the full potential for CTC analysis must include molecular analyses. For transcriptional profiling of specific individual targets analyzed by specific analytical assays, viable cells isolated from blood anticoagulated via EDTA or Citrate (ACD) can be used if processed within a short time after phlebotomy or if transported under controlled cooled conditions. Broader use of CTC enrichment, enumeration, and molecular testing, including the transcriptome, and less cost-intensive sample transport, and storage at ambient temperatures, is currently hindered by the lack of suitable sample preservation technologies.

Via the CEN/TC 140, the European Union’s HORIZON 2020 SPIDIA4P consortium project with 19 partners from 11 countries (2017–2020, www.spidia.eu) has started to develop CEN/Technical Specifications for pre-analytical workflows addressing different CTCs, exosomes, and other EVs, as well as ccfRNA applications. The consortium is working together with various other international consortia and professional societies to achieve broad consensus on these pre-analytical workflow requirements and recommendations. One key SPIDIA4P collaborator is the CANCER-ID consortium, a European consortium with 40 industry and academic partner from 14 countries and supported by the European Union’s Innovative Medicines Initiative (IMI), focusing as well on pre-analytical workflows for CTCs and other liquid biopsies (www.cancer-id.eu).

The situation is different for ccfDNA analysis. Since Streck introduced the Cell-Free DNA BCT, many other dedicated blood collection tubes with ccfDNA stabilizing reagents became commercially available. In addition to the Streck tube, the PAXgene Blood ccfDNA Tube (PreAnalytiX) and the cfD Tube (Roche) have been recently scrutinized in comparison tests and validation studies. The growing number of technical solutions, validation, and standardization efforts can be seen as an indicator that in the field of cancer, broad routine use of ctDNA to complement existing procedures is coming soon.

## Abbreviations

BCT: blood collection tube
ccfDNA: circulating cell-free DNA
cffDNA: cell-free fetal DNA
ctDNA: circulating tumor DNA
CTC: circulating tumor cells
EMT: epithelial to mesenchymal transition
EV: extracellular vesicle
gDNA: genomic DNA
NIPT: non-invasive prenatal testing
qPCR: quantitative, real-time PCR
WBC: white blood cell
